# How light, temperature, and measurement and growth [CO_2_] interactively control isoprene emission in hybrid aspen

**DOI:** 10.1093/jxb/eru443

**Published:** 2014-11-13

**Authors:** Ülo Niinemets, Zhihong Sun

**Affiliations:** ^1^Estonian University of Life Sciences, Kreutzwaldi 1, 51014 Tartu, Estonia; ^2^Estonian Academy of Sciences, Kohtu 6, 10130 Tallinn, Estonia

**Keywords:** CO_2_ response, dimethylallyl diphosphate, elevated [CO_2_], isoprene emission, light sensitivity, temperature optimum, temperature response.

## Abstract

Plant isoprene emissions have been modelled assuming independent controls by light, temperature and atmospheric [CO_2_]. However, the isoprene emission rate is ultimately controlled by the pool size of its immediate substrate, dimethylallyl diphosphate (DMADP), and isoprene synthase activity, implying that the environmental controls might interact. In addition, acclimation to growth [CO_2_] can shift the share of the control by DMADP pool size and isoprene synthase activity, and thereby alter the environmental sensitivity. Environmental controls of isoprene emission were studied in hybrid aspen (*Populus tremula* × *Populus tremuloides*) saplings acclimated either to ambient [CO_2_] of 380 μmol mol^–1^ or elevated [CO_2_] of 780 μmol mol^–1^. The data demonstrated strong interactive effects of environmental drivers and growth [CO_2_] on isoprene emissions. Light enhancement of isoprene emission was the greatest at intermediate temperatures and was greater in elevated-[CO_2_]-grown plants, indicating greater enhancement of the DMADP supply. The optimum temperature for isoprene emission was higher at lower light, suggesting activation of alternative DMADP sinks at higher light. In addition, [CO_2_] inhibition of isoprene emission was lost at a higher temperature with particularly strong effects in elevated-[CO_2_]-grown plants. Nevertheless, DMADP pool size was still predicted to more strongly control isoprene emission at higher temperatures in elevated-[CO_2_]-grown plants. We argue that interactive environmental controls and acclimation to growth [CO_2_] should be incorporated in future isoprene emission models at the level of DMADP pool size.

## Summary

Isoprene emission is typically modelled using independent controls of light, temperature, and ambient [CO_2_], assuming these are unaffected by growth [CO_2_]. We demonstrated strong interactive environmental controls on emissions, calling for profound revision of emission algorithms.

## Introduction

Isoprene as a highly reactive and the most widespread volatile molecule emitted from a series of plant species plays a major role in air quality and climate, participating in ozone and secondary organic aerosol generation (Claeys *et al.*, 2004; Fowler *et al.*, 2009; Monson *et al.*, 2012; Fineschi *et al.*, 2013; Sharkey *et al.*, 2013). The biological role of isoprene in plants is protection from abiotic stresses by serving as a membrane stabilizer under heat stress (Singsaas *et al.*, 1997; Sharkey *et al.*, 2001, 2008; Siwko *et al.*, 2007), as well as a lipid-soluble antioxidant reacting with a broad array of stress-generated reactive oxygen species and peroxidized membrane lipids (Loreto *et al.*, 2001; Affek and Yakir, 2002; Vickers *et al*., 2009*a*, *b*; Possell and Loreto, 2013).

Isoprene formation in chloroplasts from dimethylallyl diphosphate (DMADP) is catalysed by isoprene synthase (for reviews, see Li and Sharkey, 2013*b*; Rosenkranz and Schnitzler, 2013; Sharkey *et al.*, 2013), and the control of isoprene synthesis under different environmental conditions is shared between isoprene synthase and the DMADP pool size (Rasulov *et al.*, 2009*b*, 2010; Possell and Hewitt, 2011; Sun *et al.*, 2012*b*; Li and Sharkey, 2013*a*, *b*; Monson, 2013). Changes in DMADP pool size are primarily responsible for the hyperbolic increase of isoprene emission with increasing quantum flux density, while the Arrhenius-type temperature response with an optimum depends both on temperature effects on isoprene synthase activity and DMADP pool size (Rasulov *et al.*, 2009*b*, 2010; Li *et al.*, 2011; Monson *et al.*, 2012; Li and Sharkey, 2013*a*). In addition, increases of CO_2_ concentration above approximately 100–150 μmol mol^–1^ inhibit isoprene emission (Wilkinson *et al.*, 2009; Possell and Hewitt, 2011; Monson *et al.*, 2012) due to a reduced chloroplastic DMADP pool size (Rasulov *et al.*, 2009*b*; Wilkinson *et al.*, 2009; Li and Sharkey, 2013*b*).

Empirical isoprene emission models widely assume that different environmental drivers operate independently (for recent reviews, see Monson *et al.*, 2012; Grote *et al.*, 2013). While empirical models have been relatively successful in simulating isoprene emission responses to temperature and light assuming independent controls (Guenther, 1997; Guenther *et al.*, 1993, 2006), it is less clear whether an analogous addition of the [CO_2_] response (e.g. Wilkinson *et al.*, 2009) is pertinent. Based on an additive [CO_2_] response, the models have indicated that isoprene emissions will decline in the future higher atmospheric [CO_2_] conditions (e.g. Heald *et al.*, 2009; Wilkinson *et al.*, 2009). However, because the effects of environmental drivers are mediated through the DMADP pool size, the effects of certain environmental combinations can be interactive rather than additive (Rasulov *et al.*, 2009*b*, 2010; Sun *et al.*, 2012*b*; Li and Sharkey, 2013*a*, *b*).

Prediction of isoprene emissions in future conditions is further complicated by acclimation of isoprene emissions to growth [CO_2_] (i.e. the [CO_2_] at which the plant is grown). There is evidence that growth [CO_2_] can modify the instantaneous [CO_2_] response of isoprene emission and the maximum emission rate (Calfapietra *et al.*, 2007, 2008, 2013; Wilkinson *et al.*, 2009; Sun *et al.*, 2012*b*) due to changes in the isoprene synthase activity and DMADP pool size (Sun *et al.*, 2012*b*, 2013). Although the DMADP pool size is characteristically reduced in plants grown under elevated [CO_2_] (Possell and Hewitt, 2011; Sun *et al.*, 2012*b*, 2013), isoprene synthase activity is not always reduced and might compensate for the reductions in DMADP pool size (Sharkey *et al.*, 1991; Li *et al.*, 2009; Sun *et al.*, 2012*b*). Such changes in DMADP pool size and isoprene synthase activity in response to growth conditions are important as they can alter the light and temperature responses. As we have demonstrated in a previous study (Sun *et al.*, 2013), higher measurement [CO_2_] (i.e. the [CO_2_] at which the rate is measured) inhibited isoprene emission rate at temperatures of 30–35 ºC, but the [CO_2_] inhibition was lost at higher temperatures, indicating enhanced DMADP availability at higher [CO_2_]. Such an enhancement is consistent with the hypothesis that low DMADP availability at high [CO_2_] is associated with reduced chloroplastic inorganic phosphate levels due to imbalanced rates of starch and sucrose synthesis consuming triose phosphates and photosynthesis providing triose phosphates, ultimately leading to feedback inhibition of photosynthesis (Li and Sharkey, 2013*b*). In fact, feedback inhibition of photosynthetic electron transport rate and ATP synthesis rate (Sharkey, 1985; Socias *et al.*, 1993) can ultimately be responsible for the decrease in DMADP synthesis rate under high measurement [CO_2_] (Rasulov *et al.*, 2009*b*). As sucrose synthesis strongly responds to temperature (Sage and Sharkey, 1987; Li and Sharkey, 2013*b*), this frees up inorganic phosphate and releases the feedback inhibition, thereby enhancing the rate of photosynthetic electron transport, and ATP and DMADP synthesis rates. This is expected to lead to a strong interactive effect of measurement [CO_2_] and temperature on isoprene emissions that can further be modified by acclimation to elevated [CO_2_].

In this study, we asked how the instantaneous CO_2_ sensitivity of isoprene emission varied with other environmental drivers, in particular with temperature, light, and growth [CO_2_], in the strongly isoprene-emitting hybrid aspen (*Populus tremula* × *Populus tremuloides*). We tested the hypotheses that elevated-[CO_2_]-grown plants would have modified environmental responses of isoprene emission and that such modified environmental responses in plants acclimated to different [CO_2_] would represent interactive controls on isoprene emission. In this model-based analysis, we integrated data reported in our previous studies (Sun *et al.*, 2012*b*, 2013) as well as additional replicate measurements, and analysed the data from the perspective of simultaneous limitation of isoprene emission by light, temperature, and [CO_2_] under different growth [CO_2_] regimes. The results of this study will provide novel insights for developing models to predict isoprene emissions in future climates.

## Material and methods

### Plant growth and experimental treatments

In this study, we included data for four series of replicate experiments reported by Sun *et al.* (2012*b*, 2013) and an additional two series of experiments conducted according to the same protocol outlined briefly here. Two-year-old saplings of hybrid aspen (*Populus tremula L. × Populus tremuloides* Michx.) clone H200 (Rasulov *et al.*, 2009*a*, 2011; Vahala *et al.*, 2003, for details of the genotype) grown in whole plant chambers were used. Four saplings were grown at a time in a four-chamber growth/gas-exchange system (individual chamber volume 12.5 l). The [CO_2_] was maintained at an ambient level (average ± standard deviation) of 380±10 μmol mol^–1^ in chambers 1 and 3, and at an elevated level of 780±10 μmol mol^–1^ in chambers 2 and 4. The chamber air temperature was 28–30/23 °C (day/night), relative humidity was 60%, and light intensity at the top of the plants was 500–800 μmol m^–2^ s^–1^ for the 12h light period, resulting in a moderately high daily integrated growth light of 28.1mol m^–2^ d^–1^ (~70% of seasonal average daily integrated quantum flux density at a completely open location in the field) (Sun *et al*., 2012*a*, 2012*b*).

### Measurement of temperature response curves of isoprene emission

Isoprene emission measurements were conducted after 30–40 d of growth under the given conditions when the plants had filled the chambers using individual attached fully mature leaves as described in detail by Sun *et al.* (2012*b*, 2013). After moving the plant out of the chamber, the sample leaf was enclosed in a Walz GFS-3000 portable gas-exchange/chlorophyll fluorescence system equipped with an LED array/PAM fluorimeter 3055-FL (Walz GmbH, Effeltrich, Germany) and connected to a Fast Isoprene Sensor (FIS, Hills-Scientific, Boulder, CO, USA). The leaf was first stabilized at the baseline conditions (leaf temperature of 30 °C, light intensity of 500 μmol m^–2^ s^–1^, and relative air humidity of 60%). Once the steady-state gas-exchange and isoprene emission rates had been established, the temperature responses of photosynthesis and isoprene emission were measured at a moderately high light intensity of 500 μmol m^–2^ s^–1^ (growth light intensity) and a strong light intensity of 2000 μmol m^–2^ s^–1^ at the [CO_2_] of 380 and 780 μmol mol^–1^. During the measurements, the leaf temperature was increased in steps of 5 °C from 30 to 50 °C, and the values of the isoprene emission rate were recorded for 8min after the change in temperature (Sun *et al.*, 2013). This time corresponds to the duration of intermediate-length sunflecks in plant canopies (Pearcy, 1990) and, although arbitrary, standardization of the time of measurement results in a common heat dose for all plants. Such a standardization is particularly important for the higher temperatures between 45 and 50 °C that can be inhibitory for photosynthesis (Hüve *et al.*, 2006, 2011) and isoprene emission (Rasulov *et al.*, 2010, 2014*a*) such that steady-state photosynthesis and isoprene emission rates cannot be reached at these higher temperatures.

### Normalized emission rates and fitting the temperature responses of isoprene emission

To normalize the environmental responses of isoprene emission, we calculated the relative light-dependent increase of isoprene emission, *R*_L_, as:

$$R_{\text{L}}=\frac{I_{\text{2000}}-I_{500}}{I_{2000}},$$ (1)

where *I*_2000_ is the isoprene emission rate at the light intensity of 2000 μmol m^–2^ s^–1^ and *I*_500_ is that at 500 μmol m^–2^ s^–1^. Analogously, the relative temperature-dependent change in isoprene emission (*R*_T_) was calculated as:

$$R_{\text{T}}=\frac{I_{\text{T}}-I_{30}}{I_{30}},$$ (2)

where *I*_30_ is the emission rate at 30 ºC and *I*_T_ is that at temperature *T*.

The temperature response of isoprene emission rate was also fitted by an exponential function with a maximum (Copolovici *et al.*, 2005; Harley and Tenhunen, 1991):

$$I=\frac{e^{c-\Delta H_{a}/RT}}{1+e^{(\Delta ST-\Delta H_{d})/RT}},$$ (3)

where *T* is the leaf temperature in K, *R* (8.314 J mol^–1^ K^–1^) is the gas constant, *c* is the scaling factor, Δ*H*_a_ (J mol^–1^) is the activation energy, Δ*H*_d_ (J mol^–1^) is the deactivation energy, and Δ*S* (J mol^–1^ K^–1^) is the entropy term. The explained variance of temperature relationships (*r* ^2^) was in all cases >0.98. From this equation, the optimum temperature for *I*, *T*_opt_ (Niinemets *et al.*, 1999*a*), is given as:

$$T_{\text{opt}}\text{=}\frac{\Delta H_{\text{d}}}{\Delta S+R\ln\left(\frac{\Delta H_{\text{d}}}{\Delta H_{\text{a}}}-1\right)}.$$ (4)

Equation 3 is analogous to the temperature relationship of the Guenther *et al.* model (Guenther, 1997; Niinemets *et al.*, 2010; Monson *et al.*, 2012; Grote *et al.*, 2013), but we favoured it in this study to demonstrate the mechanistic connection between the parameters of the temperature relationship and *T*_opt_.

To characterize the initial increase of isoprene emission rate with increasing temperature, we also calculated the average value of Q_10_, the process rate at temperature *T*+10 ºC relative to the process rate at temperature *T*, for the temperature range 25–40 ºC using the fitted temperature response curve parameters (Eq. 3).

The temperature response of isoprene emission is a mixed response that is driven by temperature effects on the DMADP pool size (*C*_DMADP_, nmol m^–2^) and on the isoprene synthase rate constant (*k*, s^–1^):

$$I=kC_{\text{DMADP}}.$$ (5)

Implicit in Eq. 5 is that the *K*_m_ value for DMADP of isoprene synthase is large relative to the concentrations of DMADP characteristically observed in chloroplasts (Rasulov *et al*., 2009*a*, *b*, 2010) such that the rate constant, *k*, does not depend on substrate concentration over the given DMADP range. The sources of variation due to changes in *k* and *C*_DMADP_ can be separated using the response coefficient analysis (Poorter and Nagel, 2000) that provides the fractions of variance in *I* due to both of its components (Appendix 1). Using available information on *k* and *C*_DMADP_ at 30 °C (Sun *et al.*, 2012*b*), the response coefficients were calculated as described in Appendix 1. As this simplified analysis does not consider possible modifications in the temperature dependence of *k* by measurement [CO_2_], and measurement and growth [CO_2_] interaction (Appendix 1), the response coefficients were only employed to gain insight into the changes in the light sensitivity of isoprene emission.

### Data analyses

In the following, the growth [CO_2_] treatments (380 vs 780 μmol mol^–1^) are denoted as ‘ambient’ and ‘elevated’, and the measurement [CO_2_] (380 vs 780 μmol mol^–1^) as 380 and 780. Thus, in this analysis, we had four combinations of growth and measurement [CO_2_]: ambient (380), ambient (780), elevated (380), and elevated (780), and two additional combinations of the measurement light intensity: a moderately high light intensity of 500 μmol m^–2^ s^–1^ and strong light intensity of 2000 μmol m^–2^ s^–1^. The effects of combinations of [CO_2_] treatment and measurement [CO_2_] at different light intensities and temperatures were analysed by analysis of variance (ANOVA) followed by Tukey’s test (growth [CO_2_] treatments involving independent samples) and by paired-samples *t*-tests (paired comparisons between different light and measurement [CO_2_]). Correlative relationships among leaf traits were analysed by linear regressions. To compare the statistical relationships among [CO_2_] treatments at different light intensities and measurement [CO_2_], analysis of covariace (ANCOVA) was used. The separate slope ANCOVA model with the interaction term (treatment with covariate) was fitted first, followed by the common-slope model (without the interaction term) when the interaction term was statistically not significant. For all analyses, we used SPSS 17.0 (IBM SPSS Statistics), and all statistical tests were considered significant at *P*<0.05.

## Results

### Dependencies of isoprene emission rate on temperature

**Fig. 1.**
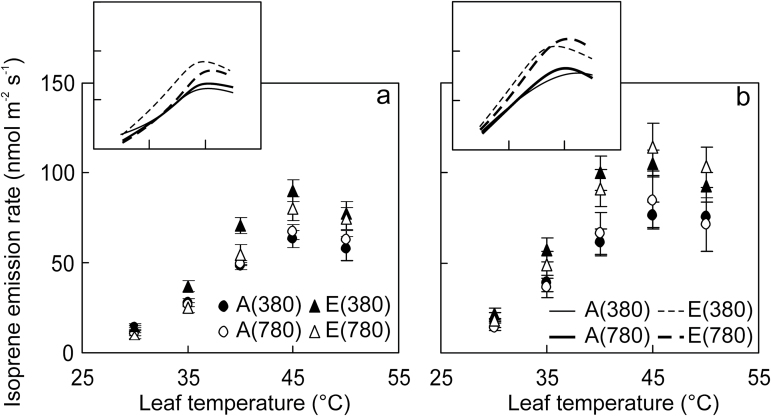
Temperature responses of isoprene emission rate in hybrid aspen leaves grown under ambient (380 μmol mol^–1^) and elevated (780 μmol mol^–1^) CO_2_ concentrations (reanalysis of the data of Sun *et al.*, 2013). Isoprene emission rate was measured both at ambient and elevated [CO_2_] and at a moderately high light intensity of 500 μmol m^–2^ s^–1^ (a) and a strong light intensity of 2000 μmol m^–2^ s^–1^ (b). A(380) and E(380) denote plants grown under ambient [CO_2_] of 380 μmol mol^–1^ and elevated [CO_2_] of 780 μmol mol^–1^, and both measured at [CO_2_] of 380 μmol mol^–1^, while A(780) and E(780) denote plants grown under ambient [CO_2_] of 380 μmol mol^–1^ and elevated [CO_2_] of 780 μmol mol^–1^, and both measured at [CO_2_] of 780 μmol mol^–1^. Reported data are averages ± standard error (SE) of 8–10 replicate leaves for each combination of environmental drivers. The insets demonstrate the curves fitted to the data (Eq. 3).

**Fig. 2.**
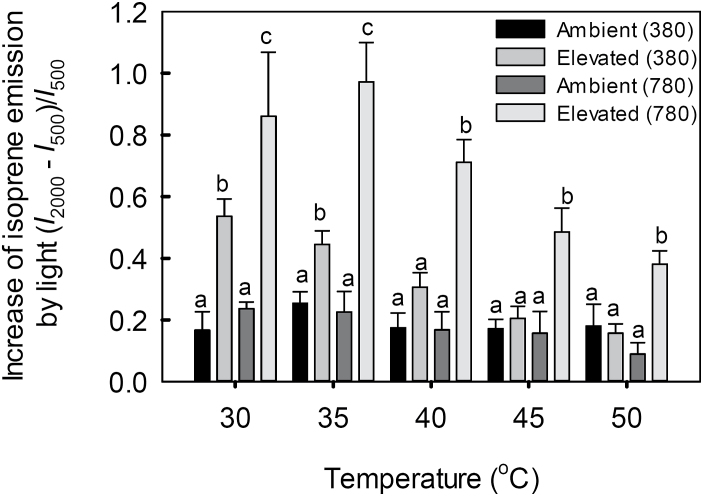
Relative increase of isoprene emission rate by increasing light level (Eq. 1) at different temperatures in hybrid aspen leaves grown under ambient [CO_2_] of 380 μmol mol^–1^ and elevated [CO_2_] of 780 μmol mol^–1^ and measured at two different CO_2_ concentrations (treatments as in Fig. 1). The increase of isoprene emission rate by increasing light was calculated as (*I*_2000_ – *I*_500_)/*I*_500_ where *I*_2000_ is the isoprene emission rate at the light intensity of 2000 μmol m^–2^ s^–1^ and *I*_500_ is that at the light intensity of 500 μmol m^–2^ s^–1^. Data are averages (+SE) of 8–10 replicate leaves. Different letters indicate significant differences among growth and measurement [CO_2_] combinations (*P*<0.05).

Increases in temperature enhanced the isoprene emission rate (*I*) up to 45–50 ºC (Fig. 1) with the optimum temperature of isoprene emission (Eq. 4) varying from 43 to 49 ºC across all the data (Table 1). Although the temperature responses were similar under the two light intensities of 500 and 2000 μmol m^–2^ s^–1^ (Fig. 1a, b), the light-dependent enhancement of *I* decreased with increasing temperature (Fig. 2). The light-dependent increase of isoprene emission rate (Eq. 1) did not depend on measurement [CO_2_] in ambient-[CO_2_]-grown plants, but in elevated-[CO_2_]-grown plants, the increase was greater at the higher measurement [CO_2_] of 780 μmol mol^–1^ than at 380 μmol mol^–1^ (Fig. 2).

**Table 1.** Average (± SE) temperature optimum of isoprene emission rate (T_opt_, Eq. 3) and average Q_10_ values in hybrid aspen leaves grown under different [CO_2_] and measured under different [CO_2_] and light intensities

[CO_2_] (μmol mol^–1^)		Light intensity (μmol m^–2^ s^–1^)			
Growth	Measurement	500	2000	500	2000
		*T*_opt_ (°C)		Q_10_	
380 (ambient)	380	45.87±0.46aA	46.19±0.32bA	3.92±0.19aA	3.77±0.05aA
380 (ambient)	780	46.80±0.46abA	45.15±0.39abB	3.83±0.06aA	4.51±0.18aB
780 (elevated)	380	45.27±0.26aA	44.50±0.38aB	5.18±0.35bA	4.18±0.18aB
780 (elevated)	780	47.58±0.30bA	46.13±0.22bB	4.57±0.40abA	4.38±0.28aA

Q_10_ is given as the process rate at temperature *T*+10 relative to the process rate at temperature *T*. It was calculated from the fitted emission vs. leaf temperature relationships as an average for the temperature range of 25–40 ºC. Means with the same lowercase letter are not significantly different (*P*>0.05) among growth and measurement [CO_2_] combinations (one-way ANOVA followed by Tukey’s test), while means with the same uppercase letter are not significantly different among different measurement light intensities (paired-samples *t*-test).

### Temperature response curve characteristics of isoprene emission in relation to growth and measurement [CO_2_] and light intensity

The optimum temperature (*T*_opt_) for isoprene emission did not depend on the measurement [CO_2_] for ambient-[CO_2_]-grown plants, but *T*_opt_ was greater at the measurement [CO_2_] of 780 μmol mol^–1^ than at 380 μmol mol^–1^ in elevated-[CO_2_]-grown plants (Table 1). *T*_opt_ was greater at a moderate light intensity of 500 μmol m^–2^ s^–1^ in all cases, except for the measurements at 380 μmol mol^–1^ in ambient-CO_2_-grown plants (Table 1). Overall, the average Q_10_ values for the temperature range of 25–40 ºC (Table 1) were greater for elevated-[CO_2_]-grown plants (Table 1). In ambient-[CO_2_]-grown plants measured at 780 μmol mol^–1^, *Q*_10_ was greater at the higher light value, while the opposite was true for elevated-[CO_2_]-grown plants measured at 380 μmol mol^–1^ (Table 1).

**Fig. 3.**
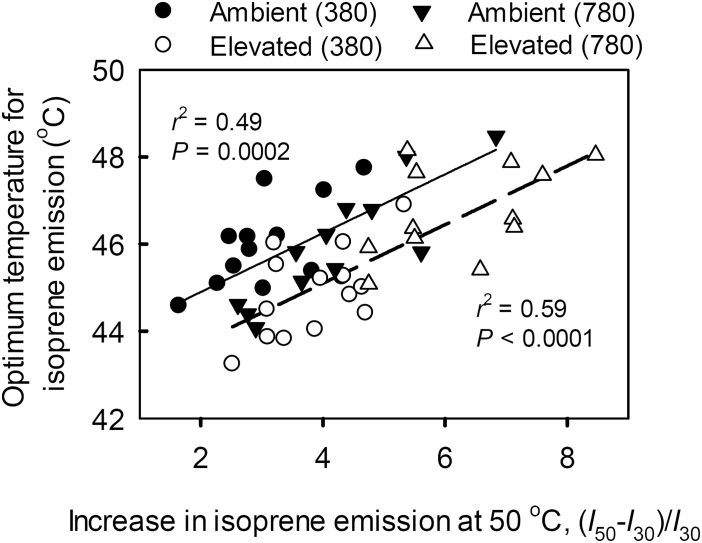
Relationships of the optimum temperature for isoprene emission (Eq. 4) with the relative increase of isoprene emission rate (*I*) with increasing temperature from 30 to 50 °C (Eq. 2) in hybrid aspen leaves grown under two different CO_2_ concentrations (ambient vs elevated) and measured at different ambient CO_2_ concentrations of 380 and 780 μmol mol^–1^, and at different light intensities of 500 and 2000 μmol m^–2^ s^–1^ (symbols for different light intensities not shown separately). Separate regression lines were fitted to the data from different growth CO_2_ treatments (*P*<0.05 for the growth CO_2_ effect according to a common-slope ANCOVA model). Table 1 shows a comparison of average *T*_opt_ values at different growth and measurement [CO_2_] conditions.

To gain insight into the sources of variation in *T*_opt_, we also analysed the correlations of *T*_opt_ with temperature response curve parameters (Eq. 3) and with traits characterizing the temperature sensitivity of emissions to lower and higher temperatures (Q_10_ and *R*_T_, Eq. 2). As isoprene synthase itself has a very high optimum temperature of around 50 ºC (Monson *et al.*, 1992; Lehning *et al.*, 1999; Rasulov *et al.*, 2010), lower *T*_opt_ values than those for isoprene synthase suggest limitation of isoprene synthesis by the DMADP pool size (Rasulov *et al.*, 2010). Accordingly, variation in *T*_opt_ at a given measurement [CO_2_] and light level should reflect differences in the heat-dependent decay of the DMADP pool size. *T*_opt_ was positively correlated with a relative increase of isoprene emission rate at 50 °C (*R*_T_, Eq. 2; Fig. 3). In this relationship, the interaction terms, *R*_T_×(growth [CO_2_]) (*P*>0.1), *R*_T_×(light intensity) (*P*>0.6) and *R*_T_×(measurement [CO_2_]) (*P*>0.7) were not statistically significant. According to the common-slope ANCOVA model, both light intensity (*P*<0.03, Table 1), and growth [CO_2_] (*P*<0.05, Fig. 3) were statistically significant factors, implying that *T*_opt_ was lower at a given *R*_T_ both at higher measurement light and in elevated-[CO_2_]-grown plants (Fig. 3), suggesting a greater control by the DMADP pool size.

In contrast to these correlations, *T*_opt_ was not correlated with the average Q_10_ for the temperature range 25–40 °C (Table 1, *r* ^2^=0.07, *P*>0.07 for all data pooled), and the correlations were much weaker for *R*_T_ values calculated for temperatures of 45 °C (*r* ^2^=0.20, *P*<0.05 for ambient-[CO_2_]-grown plants and *r* ^2^=0.10, *P*>0.1 for elevated-[CO_2_]-grown plants) and 40 °C (*r* ^2^=0.01 for ambient-CO_2_-grown and *r* ^2^=0.02 for elevated-[CO_2_]-grown plants, *P*>0.8 for both). In addition, differences in *T*_opt_ were mainly associated with differences in the deactivation energy (Δ*H*_d_, Eq. 3). Thus, the magnitude of the initial increase of isoprene emission at lower temperatures and the onset of the emission decrease at higher temperatures were essentially independent.

### Sources of variation in isoprene emission rate due to the isoprene synthase rate constant and DMADP pool size

**Fig. 4.**
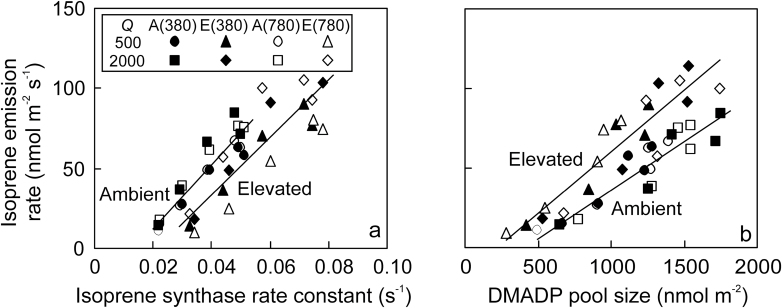
Correlations of isoprene emission rate measured under different combinations of environmental drivers [growth and measurement [CO_2_] (μmol mol^–1^) and quantum flux density (μmol m^–2^ s^–1^)] with predicted isoprene synthase rate constant (*k*, a) and isoprene substrate DMADP pool size (*C*_DMADP_, b). The isoprene emission rate is given as *kC*_DMADP_ (Eq. 5), whereas the components of *k* and *C*_DMADP_ were resolved by a modelling analysis as explained in Materials and methods. Experimental treatments were as in Fig. 1. Different data points within the given data series correspond to the average values at each temperature (the same data as in Fig. 1). Data were fitted by linear regressions separately for elevated-[CO_2_]-grown plants [*r* ^2^=0.78 for (a) and *r* ^2^=0.84 for (b)] and ambient-[CO_2_]-grown plants [*r* ^2^=0.88 for (a) and *r* ^2^=0.86 for (b)] (*P*<0.001 for all regressions).

The isoprene emission rate through the temperature range 30–50 °C increased both with the predicted isoprene synthase rate constant (*k*) and with the DMADP pool size (*C*_DMADP_, Fig. 4). According to separate slope ANCOVA analyses, the slopes of *I* versus *k* (*P*>0.5) and *I* versus *C*_DMADP_ were not significantly different among elevated- and ambient-[CO_2_]-grown plants. However, elevated-[CO_2_]-grown plants had a lower isoprene emission rate at a given *k* and higher isoprene emission rate at a given DMADP pool size (*P*<0.001 for common-slope ANCOVA analyses).

The light sensitivity of isoprene emission was positively correlated with the DMADP response coefficient across all the data, while the correlation was negative for the response coefficient for *k* (*r* ^2^=0.42, *P*<0.001 for both).

## Discussion

### Interactive light and temperature dependencies of isoprene emission

Our study highlights a complex interplay between different environmental drivers and growth [CO_2_] treatments on leaf isoprene emission, identifying three novel features of how isoprene emissions respond to light and temperature in plants grown at different [CO_2_]:

1. The optimum temperature and the initial rate of increase with temperature (Q_10_) for isoprene emission varied in dependence on light intensity and growth [CO_2_] (Fig. 1, Table 1).
2. The light sensitivity of isoprene emission, defined as the change of isoprene emission rate with increasing light level, decreased with increasing temperature (Fig. 2).
3. The light sensitivity was greater in elevated-[CO_2_]-grown plants, especially when assessed at higher [CO_2_] (Fig. 2).

We argue that these interactive effects reflect changes in the share of control of emission rates by the DMADP pool size and isoprene synthase activity. There is evidence that both instantaneous light and [CO_2_] dependencies of isoprene emission are driven primarily by light- and [CO_2_]-driven changes in the DMADP pool size (Rasulov *et al*., 2009*a*, *b*; Li *et al.*, 2011; Possell and Hewitt, 2011; Li and Sharkey, 2013*a*), while the temperature dependence is a mixed response, driven both by temperature-dependent changes in DMADP pool size and isoprene synthase activity (Rasulov *et al.*, 2010, 2011; Li *et al.*, 2011; Li and Sharkey, 2013*a*). As we demonstrated in our previous study (Sun *et al.*, 2012*b*) and confirmed by the flux control analysis (Fig. 4), elevated-[CO_2_]-grown plants had greater isoprene synthase activity but a lower DMADP pool size (Sun *et al.*, 2012*b*).

In the following, we address the facets of the isoprene emission response to these complex multifactorial environmental interactions and acclimation responses based on the immediate effects of environmental conditions on the rate of DMADP synthesis as well as growth-[CO_2_]-dependent changes in overall DMADP pool size and isoprene synthase activity. We emphasize that the responses highlighted here reflect changes in the shape of the response curves and the way the controls operate, interactively versus additively. These modifications are driven primarily by the relative share of the control by DMADP pool size and isoprene synthase activity. In addition to these modifications, environmental acclimation, e.g. such as acclimation to different growth [CO_2_] or growth temperatures, also affects the overall emission rate by altering the absolute values of isoprene synthase activity and DMADP pool size, for example through leaf structural modifications such as enhanced stacking of mesophyll cells per unit leaf area as manifested in increased leaf thickness (Sun *et al.*, 2012*b*; Rasulov *et al.*, 2014*a*).

### Modification of temperature responses of isoprene emission by light and [CO_2_]

Variations in the optimum temperatures of isoprene emission, *T*_opt_, between approximately 40 and 48 °C have been observed in several studies (e.g. Singsaas and Sharkey, 1998; Niinemets *et al.*, 1999*b*; Singsaas *et al.*, 1999; Rasulov *et al.*, 2010). However, these modifications have been difficult to explain and reproduce by models, and a constant optimum temperature of 41 °C has commonly been used in models of isoprene emission (Guenther *et al.*, 1993; Guenther, 1997; see Niinemets *et al.*, 2010, for a review). In recent modelling efforts, optimum temperature has been linked to the past weather conditions (Guenther *et al.*, 2006; Guenther *et al.*, 2012), assuming that *T*_opt_ increases as leaves acclimate to hotter temperatures, but empirical and mechanistic support for such a relationship is scarce. Our study provides important evidence that *T*_opt_ can vary in dependence on measurement light intensity and measurement and growth [CO_2_] (Table 1, Figs 1 and 3). In addition, although the steady-state *T*_opt_ for isoprene emission can be relatively low, the role of isoprene in improving heat tolerance has mainly been associated with enhanced resistance of short-term increases in leaf temperature such as observed during light flecks (Behnke *et al*., 2007, 2013; Way *et al.*, 2011; Monson *et al.*, 2013). We argue that it is the transient *T*_opt_ as estimated in our study that characterizes the leaf capacity to cope with such transient increases in leaf temperature.

What could be the mechanism for light- and [CO_2_]-dependent changes in *T*_opt_? As discussed above, the temperature optimum for isoprene synthase is characteristically significantly higher than that for the DMADP pool size, suggesting that variation in *T*_opt_ with varying measurement and growth [CO_2_] and light level should be driven primarily by changes in the DMADP pool size. This reasoning is supported by the increase in *T*_opt_ with the temperature sensitivity of isoprene emission, (*I*_50_ – *I*_30_)/*I*_30_ (Fig. 3). The temperature sensitivity, (*I*_50_ – *I*_30_)/*I*_30_, itself depends both on temperature effects on isoprene synthase activity and DMADP pool size, but provided *T*_opt_ is less than the optimum for isoprene synthase activity, the way this characteristic is correlated with *T*_opt_ depends on the extent to which isoprene emission is controlled by the DMADP pool size at higher temperatures. Thus, a greater *T*_opt_ at a given value of (*I*_50_ – *I*_30_)/*I*_30_ in ambient-[CO_2_]-grown plants (Fig. 3) is in agreement with their greater DMADP pool size at the given isoprene synthase activity (Sun *et al.*, 2012*b*).

Leaves grown and measured at the higher [CO_2_] of 780 μmol mol^–1^ had both a greater *T*_opt_ (Fig. 1, Table 1) and (*I*_50_ – *I*_30_)/*I*_30_ (Fig. 3). In fact, as much of the carbon released in heat-stressed leaves is derived from ‘old’ carbon sources, in particular from starch hydrolysis (Sharkey and Yeh, 2001; Fortunati *et al.*, 2008), this strong enhancement might reflect more readily available alternative carbon sources for DMADP formation in elevated-[CO_2_]-grown plants consistent with their greater starch and soluble sugar content (Sun *et al.*, 2012*b*, 2013). However, we cannot currently rule out improved heat resistance of isoprene synthase in elevated-[CO_2_]-grown plants. Although isoprene synthase is operationally a soluble enzyme, it is strongly pH dependent (for reviews, see Rajabi Memari *et al.*, 2013; Rosenkranz and Schnitzler, 2013). Increased chloroplast membrane leakiness at high temperatures (Schrader *et al.*, 2004; Wise *et al.*, 2004) is expected to reduce stromal pH, and thus isoprene synthase might increasingly operate outside its optimum pH range. As growth under elevated [CO_2_] results in more heat-stable membranes in hybrid aspen (Sun *et al.*, 2013), the onset of the reduction in isoprene synthase activity due to chloroplast membrane leakiness might have shifted to higher temperatures in elevated-[CO_2_]-grown plants. In fact, the response coefficient analysis based on constant isoprene synthase characteristics (Appendix 1) suggested that isoprene synthase limited the flux at higher temperatures less in elevated-[CO_2_]-grown plants than in ambient-[CO_2_]-grown plants (data not shown). We argue that additional studies are needed that explicitly characterize the isoprene synthase temperature dependencies in plants grown under different [CO_2_] conditions.

The explanation based on DMADP control of *T*_opt_ also does not explain why *T*_opt_ was greater at a lower light intensity across the treatments and at a given (*I*_50_ – *I*_30_)/*I*_30_ (Table 1). Stronger activation of alternative sinks for DMADP under high light and temperature such as for the synthesis of photoprotective carotenoids, in particular, xanthophyll cycle carotenoids (Havaux and Tardy, 1996; Havaux and Niyogi, 1999), could provide a possible explanation. Xanthophylls (oxygenated carotenoids) and non-oxygenated carotenoids and tocopherols (vitamin E) play an important role in maintaining the integrity of the photosynthetic membranes under oxidative stress that typically occurs both under heat and high light (Singsaas *et al.*, 1997; Vickers *et al.*, 2009*a*; Loreto and Schnitzler, 2010; Velikova *et al.*, 2011). Recent data demonstrate that chloroplastic synthesis of higher-molecular-mass isoprenoids can operate at rates high enough to compete for DMADP at the level of geranyl diphosphate (GDP) synthesis (Ghirardo *et al.*, 2014; Rasulov *et al.*, 2014*b*). In fact, due to a lower *K*_m_ for DMADP of GDP synthases than that for isoprene synthase (reviewed by Rajabi Memari *et al.*, 2013), activation of higher isoprenoid synthases and a concomitant reduction in the DMADP pool can have significant effects on isoprene synthesis, while larger isoprenoid synthesis still proceeds with a maximum rate. Of course, none of these explanations rules out the effect of heat stress *per se*, in particular under high light, on the observed patterns.

Differences in average Q_10_ values among the measurement light intensities for ambient-[CO_2_]-grown plants measured at 780 μmol mol^–1^ and for elevated-[CO_2_]-grown plants measured at 380 μmol mol^–1^ (Table 1) further highlight the fact that light and temperature controls can interact at moderately high temperatures as well. In the case of ambient-[CO_2_]-grown plants, enhanced Q_10_ at higher measurement light (Table 1) is indicative of enhancement of the DMADP pool size by increased light level, reducing the imbalance between isoprene synthase activity and DMADP pool size (see also the discussion below for light sensitivity). In contrast, lower Q_10_ in elevated-[CO_2_]-grown plants at higher light similarly to lower *T*_opt_ (Table 1) suggests that the activation of alternative DMADP sinks at higher light can already occur at moderately high temperatures. Clearly, these data suggest that the interactive effects of [CO_2_] and light on the temperature response of isoprene emission vary for high (characterized by *T*_opt_) and moderate (characterized by average Q_10_ value for the temperature range 25–40 °C) leaf temperatures.

### Altered light sensitivity of isoprene emission under different temperatures

The enhanced light sensitivity of isoprene emission in elevated-[CO_2_]-grown plants is in agreement with experimental observations on their lower DMADP pool size and greater isoprene synthase activity. Given the smaller DMADP pool size, which strongly curbs isoprene emission, any increase in DMADP pool size at higher light readily results in a higher isoprene synthesis rate (Fig. 2). This response was particularly strong at a higher measurement [CO_2_] (Fig. 2), possibly indicating a lower initial DMADP pool size and stronger control of the emission flux by DMADP under such conditions, as discussed above. Although the *K*_m_ value of isoprene synthase for DMADP is large (Rasulov *et al*., 2009*a*, 2011, 2014*b*), a larger pool of DMADP relative to isoprene synthase activity can result in an increasingly non-linear Michaelis–Menten-type hyperbolic response (Rasulov *et al*., 2009*a*, 2014*b*), reducing the increase of isoprene emission for a given increase of DMADP pool size.

Although the light enhancement of isoprene emission became weaker with increasing temperature, the stronger light enhancement in elevated-[CO_2_]-grown plants under high measurement [CO_2_] was maintained over the entire temperature range. We suggest that these patterns result from multiple mechanisms operating at different parts of the temperature response of light sensitivity. First, the increase in temperature is initially associated with enhanced DMADP synthesis rate (Rasulov *et al.*, 2010; Li *et al.*, 2011). This reduces the DMADP limitation of isoprene emission at lower light at higher temperature. Secondly, increases in temperature enhance isoprene synthase activity, making isoprene synthase less sensitive to the DMADP pool size (Rasulov *et al.*, 2010).

Given these modifications, it is still puzzling why the light sensitivity of isoprene emission remained greater in elevated-[CO_2_]-grown leaves under high measurement [CO_2_] and high temperature (Fig. 2). This response might initially seem counterintuitive as it suggests a more enhanced DMADP pool size in elevated-[CO_2_]-grown leaves under high measurement [CO_2_]. However, heat-depressed quantum yield of photosynthesis and photosynthetic electron transport as observed by Sun *et al.* (2012*b*), especially under high light, can be responsible for curtailed enhancement of DMADP for isoprene synthesis in the case of ambient-[CO_2_]-grown plants. This, combined with the lower contribution of alterative carbon sources as (see Sun *et al.,* 2013 for a discussion) can be responsible for enhanced light sensitivity of isoprene emission, similarly to enhanced temperature stability (Table 1).

## Conclusions

Our study highlights a number of important differences among temperature responses under different growth [CO_2_] treatments and under different measurement [CO_2_] and light intensities that collectively suggest that the effects of environmental drivers interactively affect isoprene emission at the level of the DMADP pool size. Thus, future models should focus on predicting integrated environmental controls on DMADP pool size rather than considering each environmental driver independently of others. Several semi-mechanistic models have recently been put forward that link isoprene emissions to photosynthetic electron flow and isoprene synthase activity (Niinemets *et al.*, 1999*b*; Arneth *et al.*, 2007; Grote *et al.*, 2014; Morfopoulos *et al.*, 2014). These models do not yet have the capacity to predict changes in DMADP pool size, and thus application of these models depends critically on our ability to predict the environmental controls on photosynthetic electron transport and partitioning of the electron flow between different electron-consuming sinks. Nevertheless, recent semi-mechanistic models do a good job in phenomenologically capturing several of the interactive environmental responses (Grote *et al.*, 2014; Morfopoulos *et al.*, 2014).

The study further highlights the important interactive effects of acclimation to growth [CO_2_] on isoprene light and temperature responses. Consideration of such effects in models again requires understanding of growth [CO_2_] effects on isoprene synthase activity, changes in DMADP partitioning between isoprene synthesis and larger molecular mass isoprenoids, and possible modifications in isoprene synthase temperature responses. Process-based simulation of the competition for DMADP by isoprene synthase and geranyl diphosphate synthase might be particularly difficult, although linking GDP synthesis to carotenoid turnover rate as driven by photo-inhibition and heat stresses (Ramel *et al.*, 2012; Havaux, 2013) can be a promising way to link isoprene emissions to stress and long-term environmental conditions. Nevertheless, there appears to be a large variation among species in their acclimation capacity to growth [CO_2_] (Wilkinson *et al.*, 2009; Sun *et al.*, 2012*b*). We suggest that more experimental work with different model species grown under different [CO_2_] regimes is needed to gain insight into the factors controlling the partitioning of DMADP among isoprene and other competing pathways. Such an understanding is crucial for realistic parameterization of interactive environmental control of isoprene emission under global change.

## Abbreviations:

ANOVA: analysis of variance
ANCOVA: analysis of covariance
DMADP: dimethylallyl diphosphate
GDP: geranyl diphosphate.

## Appendix 1. Response coefficients for modelling temperature responses

Assuming that the temperature response of the isoprene synthase rate constant (*k*, s^−1^) is the inherent property of isoprene synthase, we used the shape of the temperature relationship of *k* analogous to Eq. 3 previously estimated for hybrid aspen isoprene synthase (Rasulov *et al.*, 2010) and scaled it to the measurements of *k* observed at different combinations of growth and measurement [CO_2_] at 30 °C (data of Sun *et al.*, 2012*b*). After scaling, *k* was predicted for each individual leaf through the entire temperature response, and the modelled DMADP pool size (C_DMADP_, nmol m^−2^) was calculated as *I*/*k*.

The sources of variation in isoprene emission rate from the rate *I*_1_ to the rate *I*_2_ in response to environmental variation can be partitioned among *k* and *C*_DMADP_ using the response coefficient analysis (Poorter and Nagel, 2000). The relative change of isoprene emission *I*_1_/*I*_2_ is given as:

$$\frac{I_{1}}{I_{2}}=\frac{k_{1}C_{\text{DMADP,}1}}{k_{2}C_{\text{DMADP,}2}}.$$ (6)

Natural logarithmic transformation of both sides of the equation gives:

$$\begin{array}{l} \left(\ln I_{1}-\ln I_{2}\right)=\left(\ln k_{1}-\ln k_{2}\right)\\ \;+\left(\ln C_{\text{DMADP,}1}-\ln C_{\text{DMADP,}2}\right), \end{array}$$ (7)

and dividing by (ln*I*_1_ – ln*I*_2_) yields:

$$1=\frac{\left(\ln k_{1}-\ln k_{2}\right)}{\left(\ln I_{1}-\ln I_{2}\right)}+\frac{\left(\ln C_{\text{DMADP,}1}-\ln C_{\text{DMADP,}2}\right)}{\left(\ln I_{1}-\ln I_{2}\right)},$$ (8)

where the first part of the equation provides the fraction of variance in *I* that is due to the variation in isoprene synthase rate constant and the second part provides the fraction of variance that is due to the variation in DMADP pool size. Thus, the response coefficients for *k* and DMADP pool size characterize the sensitivity of isoprene emission to variations in these drivers at the given isoprene synthesis rate (mathematically, the response coefficient for *k* can also be defined as $\frac{dk}{k}/\frac{dI}{I}$). The concept of response coefficient is analogous to flux control coefficients in metabolic flux control analysis (Woodrow and Mott, 1993; Stitt and Schulze, 1994). The response coefficients were calculated through the temperature dependence of isoprene emission relative to the values at 30 °C (*k*_2_, *C*_DMADP,2_, and *I*_2_).
